# Enhanced Electrical Conductivity of Sb_2_S_3_ Thin Film via C_60_ Modification and Improvement in Solar Cell Efficiency

**DOI:** 10.1002/gch2.201800108

**Published:** 2019-02-27

**Authors:** Chunsheng Guo, Jingwei Chen, Gang Li, Xiaoyang Liang, Weidong Lai, Lin Yang, Yaohua Mai, Zhiqiang Li

**Affiliations:** ^1^ Hebei Key Laboratory of Optic‐Electronic Information and Materials College of Physics Science and Technology Hebei University Baoding 071002 China; ^2^ Institute of New Energy Technology Jinan University Guangzhou 510632 China

**Keywords:** antimony sulfide, C_60_ modification, lattice distortion, photodetectors, solar cells, thin films

## Abstract

Sb_2_S_3_ has attracted great research interest very recently as a promising absorber material for photoelectric and photovoltaic devices because of its unique optical and electrical properties and single, stable phase. However, the intrinsic high resistivity property of Sb_2_S_3_ material is one of the major factors restricting the further improvement of its application. In this work, the C_60_ modification of Sb_2_S_3_ thin films is investigated. The conductivity of Sb_2_S_3_ thin films increases from 4.71 × 10^−9^ S cm^−1^ for unmodified condition to 2.86 × 10^−8^ S cm^−1^ for modified thin films. Thin‐film solar cells in the configuration of glass/(SnO_2_:F) FTO/TiO_2_/Sb_2_S_3_(C_60_)/Spiro‐OMeTAD/Au are fabricated, and the conversion efficiency is increased from 1.10% to 1.74%.

## 1 Introduction

In recent years, a great deal of effort has been devoted to explore the application of novel materials such as Cu_2_ZnSn(S,Se)_4_,^1^, ^2^ SnS,^3^ CuSb(S,Se)_2_,^4^, ^5^ GeSe,^6^ Sb_2_(S,Se)_3_ 7, 8, 9, 10, 11 for light absorption materials for solar energy conversion. Among them, antimony sulfide (Sb_2_S_3_) is a binary semiconductor compound with a single stable phase, which could avoid the formation of other secondary phases.^12^ In particular, Sb_2_S_3_ received considerable attention for light absorber material in solar cells due to its suitable bandgap of 1.7–1.8 eV with high absorption coefficient of 10^5^ cm^−1^ in the visible range, abundant and environmental friendly compositional elements, and excellent air stability.^13^, ^14^, ^15^ This bandgap allows its application as absorber in single‐junction solar cell or in top cell of the multijunction tandem photovoltaic device.

The carrier diffusion length of Sb_2_S_3_ could reach hundreds of nanometers,^14^ which allowed its application in both sensitized mesoporous device structure and planar heterojunction solar cell configuration. Sb_2_S_3_ was employed as semiconductor sensitizer in mescoscopic solar cells in 2009, where the extremely thin Sb_2_S_3_ absorber (between several nanometers and several tens of nanometers) was made using chemical bath deposition (CBD), and a conversion efficiency of 3.37% was obtained.^16^ Choi et al. reported conversion efficiency as high as 7.5% in 2014, by post‐treatment for reducing the trap sites in Sb_2_S_3_.^17^

On the other hand, Sb_2_S_3_‐based planar heterojunction solar cells also gained great progress in device fabrication, device structure improvement, and so on. Compared to the several nanometers‐thick absorber in Sb_2_S_3_‐sensitized solar cells, the thickness of Sb_2_S_3_ absorber in planar heterojunction solar cell was in scale of hundreds of nanometers. Both the surface defect and bulk defect played critical roles in dictating solar cell device performance. The intrinsic high resistivity property of Sb_2_S_3_ material was one of the major factors restricting the further improvement of device performance.^18^ Introducing other elements as dopant to modify the host system or forming ternary alloyed compounds was an efficient route to tailor the electronic band dispersion, adjust the carrier concentration, or even vary the conductivity type of the Sb_2_S_3_‐based semiconductor. Very recently, Tang et al. reported controllable sulfur vacancy defect by introducing Zn ion into the films. The behavior of electron concentration in Sb_2_S_3_ layer was observed to increase with sulfur vacancy defects, which resulted in reduced series resistance and increased recombination resistance for the Sb_2_S_3_ thin film solar cells.^19^

However, the incorporation of metal or non‐metal element doping or formation of alloy with other material, usually presented in the final product in the form of ion, led to the distortion of lattice structure and induced parasitic effect to the Sb_2_S_3_. For example, the bismuth dopant decreased the surface state of Sb_2_S_3_ crystals and caused the variation of band dispersion and electronic structure of Sb_2_S_3_.^20^ Carbon was also employed as dopant in the Sb_2_S_3_ thin layers due to its very high solubility and very low diffusion coefficient. Cardenas et al. investigated the effect of surface modification of arc‐deposited carbon layer on Sb_2_S_3_ thin films. The resistivity of Sb_2_S_3_ thin films was reduced from 10^8^ Ω cm for as‐prepared condition to 10^2^ Ω cm for post‐treated thin films, while the bandgap for carbon‐doped sample was kept nearly values with the undoped crystalline Sb_2_S_3_ thin films.^18^ The investigation of the carbon doping on Sb_2_S_3_ was not enough.

In this work, we explore the C_60_‐modified Sb_2_S_3_ thin films by spin coating. The presence of C_60_ was checked by scanning electron microscopy (SEM) and Raman spectra. Both of the longitudinal and transverse conductivity of the Sb_2_S_3_ layer with and without C_60_ modification was analyzed. Planar solar cells in configuration of glass/SnO_2_:F (FTO)/TiO_2_/Sb_2_S_3_(C_60_)/Spiro:OMeTAD/Au were fabricated. The solar cells employing C_60_‐modified Sb_2_S_3_ absorber show better short‐circuit current, fill factor (FF), and conversion efficiency. Furthermore, the physical mechanism behind this improvement was discussed.

## 2 Results and Discussion

**Figure 1:**
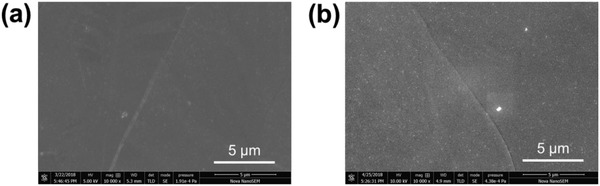
Plan‐view SEM images of the Sb_2_S_3_ thin films, a) bare Sb_2_S_3_ and b) C_60_‐modified Sb_2_S_3_ thin films.

**Figure 2:**
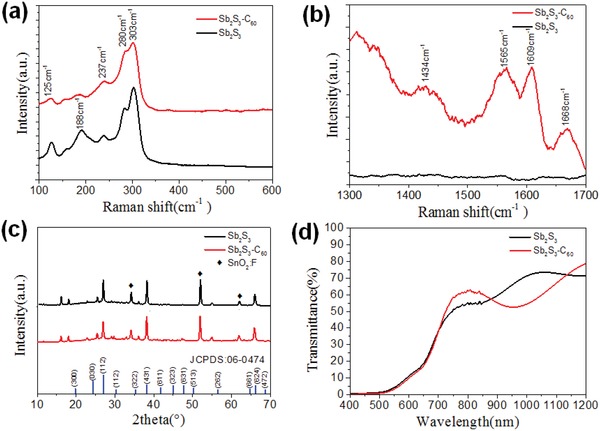
Raman spectra of Sb_2_S_3_ for the wavenumber range of 150–600 and 1300–1700 cm^−1^. a) Raman spectra of Sb_2_S_3_ and C_60_‐modified Sb_2_S_3_ in the wavenumber range of 150–600 cm^−1^. b) Raman spectra of Sb_2_S_3_ and C_60_‐modified Sb_2_S_3_ in the wavenumber range of 1300–1700 cm^−1^. c) XRD patterns of the Sb_2_S_3_ thin films with and without C_60_ modification. d) Transmittance spectra of the Sb_2_S_3_ thin films with and without C_60_ modification.

**Figure 3:**
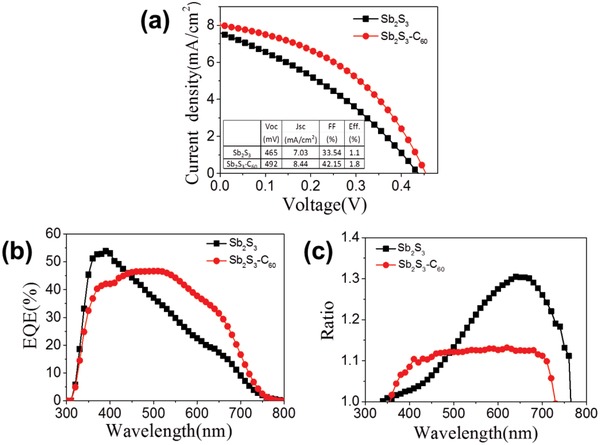
a) *J*–*V* curves of Sb_2_S_3_ thin film solar cells with and without C_60_ modification. b) EQE spectra of Sb_2_S_3_ thin film solar cells with and without C_60._ c) The ratio of EQE (−0.2 V)/EQE (0 V).

The surface morphologies of Sb_2_S_3_ thin films with and without C_60_ modification were characterized by SEM. As shown in **Figure** **1**, the Sb_2_S_3_ layer was smooth and uniform without any pinhole or particle agglomeration that could be observed, which was in agreement with the previous reports. A large number of small bright dots were observed from the SEM image of the C_60_‐modified Sb_2_S_3_ thin films. In order to characterize the small bright spots on the film that were caused by C_60_ modification, we carried out the Raman spectra measurement in the wavenumber range of 150–600 and 1300–1700 cm^−1^, respectively. As show in **Figure** **2**a, in the range between 150 and 600 cm^−1^, both of the Raman spectra exhibited similar behaviors, and four peaks at 188, 237, 280, and 303 cm^−1^ could be observed. The peaks centered at 188, 237, 280, and 303 cm^−1^ could correspond to the vibration of the Sb—Sb bond for Sb_2_S_3_ structural units, to the antisymmetric S—Sb—S vibration, the antisymmetric vibrations of Sb—S stretching in pyramidal symmetry, and the symmetric stretching of the Sb—S structural units, respectively.^21^ On the contrary, the Raman spectra in the range of 1300–1700 cm^−1^ displayed very different behaviors (Figure 2b). The Raman spectrum of bare Sb_2_S_3_ sample was smooth and no obvious peaks could be observed, while that of C_60_‐modified sample showed peaks at 1609, 1668, 1565, and 1434 cm^−1^, respectively. The peak centered at 1609 and 1668 cm^−1^ was corresponding to the vibration of H_g_ symmetry in the C_60_ unit.^22^, ^23^ The peaks centered at 1434 and 1565 cm^−1^ could be indexed to the A_g_ and H_g_ vibration mode of C_60_.^24^ This result suggested the presence of C_60_ in the Sb_2_S_3_ films. It should be noted that the X‐ray diffraction (XRD) patterns of bare and C_60_‐modified Sb_2_S_3_ thin films showed nearly the same behavior with each other. As shown in Figure 2c, both the XRD patterns of the bare and C_60_‐modified Sb_2_S_3_ films could be indexed to the orthorhombic structure of Sb_2_S_3_ (JCPDS: 06‐0474), and no other impurity phase or any shift could be observed. This behavior was much different from the case of metal or non‐metal element doping in the Sb_2_S_3_ thin films.^19^, ^25^ The lattice parameters *a*, *b*, and *c* of the bare Sb_2_S_3_ film were calculated to be 4.722, 5.695, and 15.681 Å, respectively. On the contrary, the parameters for the C_60_‐modified Sb_2_S_3_ film were 4.725, 5.691, and 15.673 Å, respectively. In addition, the transmittance spectra of the C_60_‐modified Sb_2_S_3_ film was basically the same as that of bare Sb_2_S_3_ film (Figure 2d). These results hinted that the presence of C_60_ did not induce any lattice distortions for Sb_2_S_3_ thin films. In addition, the optical bandgap of the unmodified Sb_2_S_3_ and C_60_‐modified Sb_2_S_3_ films was calculated to be about 1.76 eV, obtained from the optical transmittance spectra, which was very close to the values obtained by external quantum efficiency (EQE) curves (**Figure** **3**b).

**Figure 4:**
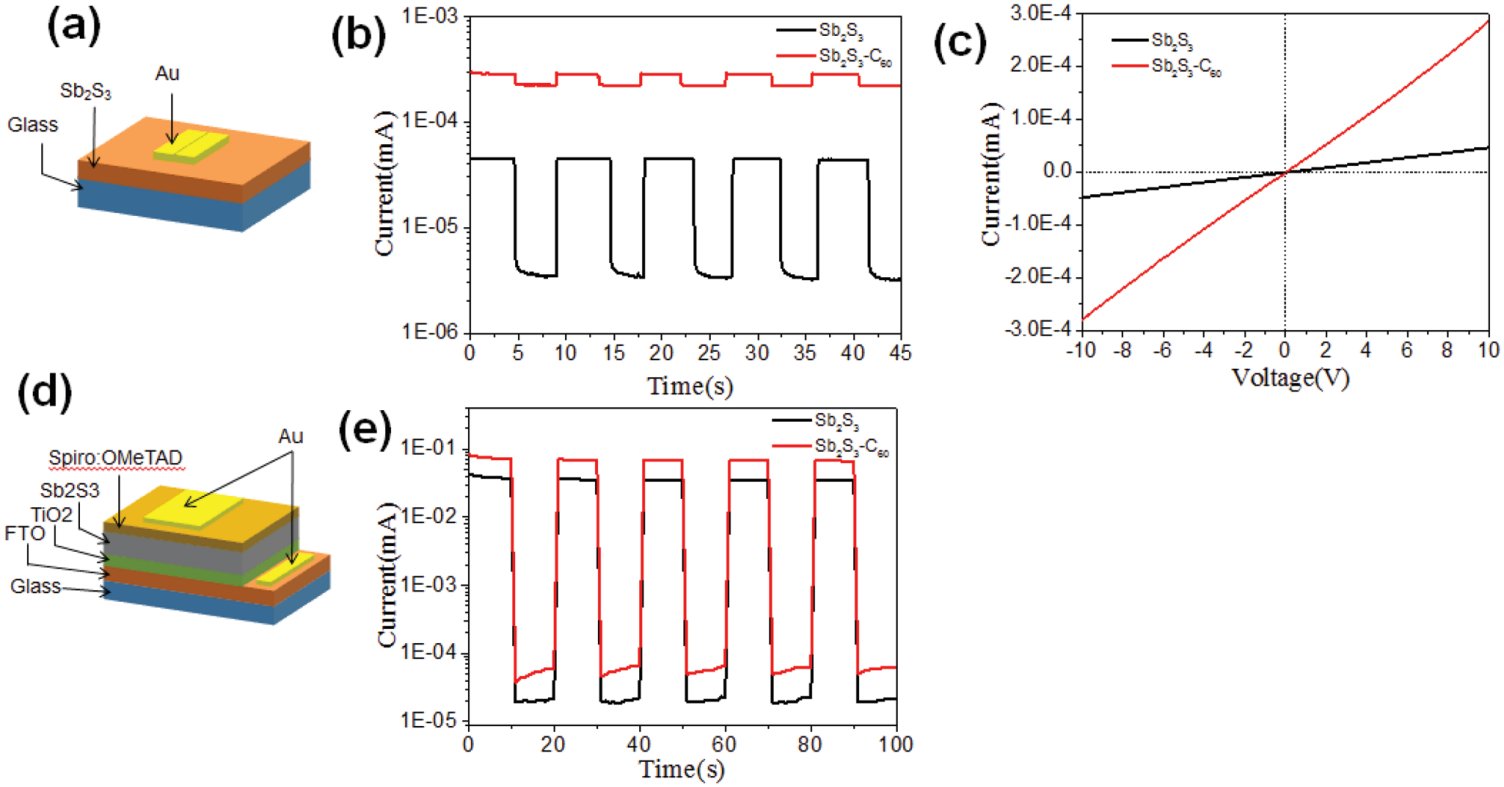
a) Schematic diagram of the transverse conductivity measurement of the Sb_2_S_3_ thin films. b) Transverse photocurrent response of Sb_2_S_3_ thin films on glass substrate with and without C_60_ modification. c) Transverse *I*–*V* curve of the Sb_2_S_3_ thin films with and without C_60_ modification. d) Schematic diagram of the longitudinal structure of the Sb_2_S_3_ thin film solar cell. e) Longitudinal photocurrent response of Sb_2_S_3_ thin film solar cells with and without C_60_ modification.

We further characterized the effect of C_60_ modification on the photoconductivity of Sb_2_S_3_ thin film. Both the transverse and longitudinal optical flow responses of the bare and C_60_‐modified Sb_2_S_3_ thin films were measured in a certain time domain (**Figure** **4**). In the case of transverse photocurrent response measurement, a Sb_2_S_3_ film with or without C_60_ was spin‐coated directly onto the FTO glass substrate as the photon‐absorber layer, followed by the thermal evaporation of a 50 nm thick gold electrode on the Sb_2_S_3_ film. The device structure of glass/Sb_2_S_3_(C_60_)/Au is formed as shown in Figure 4a.^26^ Figure 4b exhibited the light and dark transverse photocurrent responses of the bare and C_60_‐modified Sb_2_S_3_ thin films as functions of time, where the photo current was generated with irradiation of a white LED with a power density of 5 mW cm^−2^. The photocurrent of the bare and C_60_‐modified Sb_2_S_3_ thin films exhibited good repeatability. The photocurrent and dark current of bare Sb_2_S_3_ thin film was 4.53 × 10^−5^ and 3.18 × 10^−6^ mA, respectively. The transverse electrical conductivity of the bare Sb_2_S_3_ thin film was low (4.71 × 10^−9^ and 3.43 × 10^−10^ S cm^−1^) and consistent with the values in previous references. In addition, the C_60_‐modified Sb_2_S_3_ thin film and bare Sb_2_S_3_ thin film were subjected to current density–voltage (*J*–*V)* test under light illumination, as shown in Figure 4c. However, the C_60_‐modified Sb_2_S_3_ thin film showed higher conductivity (2.86 × 10^−8^ and 2.24 × 10^−8^ S cm^−1^) both under light illumination and dark conditions. This was also confirmed by the *J*–*V* curves in Figure 4c.

For longitudinal photoconductivity measurement, we fabricated the Sb_2_S_3_‐based devices in configuration of glass/FTO/TiO_2_/Sb_2_S_3_(C_60_)/Spiro‐OMeTAD/Au. In the device shown in Figure 4d, the light passed through glass, FTO, and TiO_2_ layer, and was absorbed in the Sb_2_S_3_ layer, resulting in the generation of carriers. The photon‐generated carriers were extracted by TiO_2_ and Spiro‐OMeTAD layers and collected by FTO and gold contact. Figure 4e displays the longitudinal photocurrent responses of the device with bare or C_60_‐modified Sb_2_S_3_ films as a function of time. It was observed that both samples exhibited good repeatability and stability as the transverse photocurrent response spectra. Both dark currents were low but the photocurrent was much different. The photocurrent of the bare Sb_2_S_3_ sample was around 40 µA, while that of the C_60_‐modified Sb_2_S_3_ sample was higher than 70 µA under light condition. This result suggested that both the transverse and longitudinal photoconductivity of the Sb_2_S_3_ thin films could be improved by C_60_ modification.^26^, ^27^

The photovoltaic characteristics of the solar cells, in superstrate configuration, with bare Sb_2_S_3_ and C_60_‐modified Sb_2_S_3_ absorber layers, respectively, were measured under AM 1.5G illumination. The *J*–*V* curves of the devices are shown in Figure 3a. The solar cell with bare Sb_2_S_3_ absorber exhibited an open‐circuit voltage (*V*_OC_) of 0.465 V, short current density (*J*_SC_) of 7.03 mA cm^−2^, FF of 33.54%, and a power conversion of 1.10%, respectively. The cell with C_60_‐modified Sb_2_S_3_ absorber showed a power conversion of 1.75% with a *V*_oc_ of 0.492 V, *J*_sc_ of 8.44 mA cm^−2^, and FF of 42.15%. As shown in Figure 3b, the edge of EQE spectra of the Sb_2_S_3_ and C_60_‐modified Sb_2_S_3_ solar cells were about 750 nm, but the plateau region was different from each other. The EQE spectra of the Sb_2_S_3_ solar cells sharply decreased at about 400 nm, which could be ascribed to the incomplete collection of photogenerated carriers in the solar cell. In the contrast, the plateau region of EQE for C_60_‐modified Sb_2_S_3_ solar cells was beyond 400 nm, indicating that the presence of C_60_ can effectively enhance the collection length of photogenerated carriers in the device. Furthermore, the biased EQE measurement in Figure 3c exhibited higher EQE values under −0.2 V bias voltage, suggesting an enhanced carrier collection.^28^, ^29^

**Figure 5:**
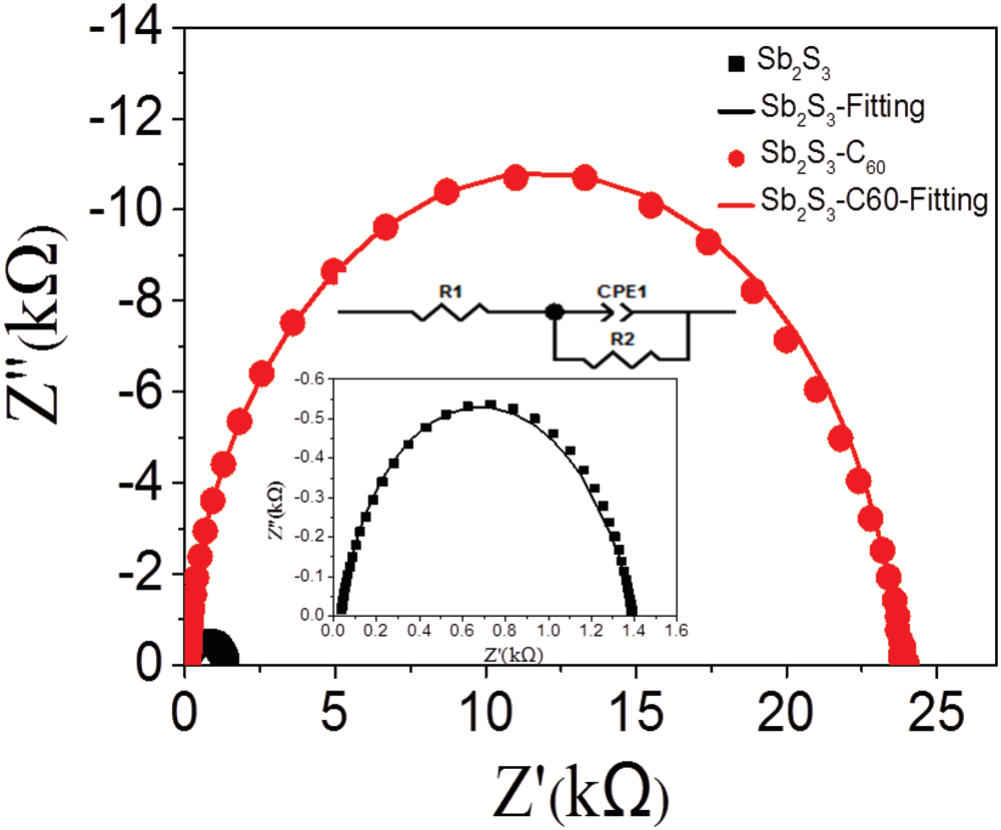
EIS of the device based on Sb_2_S_3_ and Sb_2_S_3_‐C_60_ absorber layers. The solid lines are fitted results, and the inset is the equivalent circuit.

To further explore the charge transport characteristics of the device, we performed the electrochemical impedance spectroscopy (EIS) measurement. The impedance spectra of the devices were recorded at a potential of 0 V at frequencies ranging from 1 Hz to 0.1 MHz. The Nyquist plots are shown in **Figure** **5**, and the inset is the equivalent electrical circuit model for the Sb_2_S_3_‐based thin film solar cell. The equivalent circuit diagram consisted of a series connection of a resistor (R_1_), a parallel combination of a constant phase element (CPE1), and a resistor (R_2_).^30^, ^31^, ^32^ In this experiment, the resistor R_1_ was related to the internal series resistance of the device, and R_1_ was the starting point of the real part in the Nyquist plots. R_2_ and CPE1 were associated with the interface of the absorber layer and the TiO_2_ layer. CPE1 can be represented by a capacitor (CPE1‐T) and a nonhomogeneity constant (CPE1‐P). The fitting results for these parameters are shown in **Table** **1**. The value of R_1_ for the solar cell with bare Sb_2_S_3_ absorber was 33.23 Ω cm^2^, while that value for the device with C_60_‐modified Sb_2_S_3_ absorber decreased to 9.22 Ω cm^2^. Moreover, the values for the composite resistors R_2_ were raised from untreated 1358 to 23680 Ω cm^2^ after modification. The results indicated that the modification of C_60_ on Sb_2_S_3_ facilitated charge transport from absorber to the TiO_2_ layer with less recombination.^33^, ^34^, ^35^

**Table 1:** Fitted parameters of the equivalent circuit from the EIS measurements

Absorber	R_1_ [Ω cm^2^]	CPE1‐T [10^−8^ F cm^−2^]	CPE1‐P [F cm^−2^]	R_2_ [Ω cm^2^]
Sb_2_S_3_	33.23	7.13	0.85	1358
Sb_2_S_3_‐C_60_	9.22	6.49	0.94	23680

## 3 Conclusion

In summary, the addition of C_60_ into the solution‐processed Sb_2_S_3_ thin films enhanced the transverse and longitudinal conductivity of the Sb_2_S_3_ films. The crystallinity and optical properties exhibited nearly the same behavior for the Sb_2_S_3_ films with and without C_60_ modification. The presence of C_60_ can effectively improve the thin film conductivity and facilitate the collection of photogenerated carriers in the absorber layer. Thus, the Sb_2_S_3_‐based planar heterojunction solar cells exhibited higher conversion efficiency after C_60_ modification. EIS measurement showed that the C_60_‐modified Sb_2_S_3_ thin film solar cell had a smaller series resistance and less interface recombination.

## 4 Experimental Section

The Sb_2_S_3_ thin film was deposited by spin coating on a commercial FTO glass substrate covered with a 50 nm thick TiO_2_ layer. The TiO_2_ layer was deposited by spin coating at 5000 rpm for 30 s and annealing in a muffle furnace at 500 °C for 60 min. The Sb_2_S_3_ precursor solution was prepared as follows: 1.0 mmol of Sb_2_O_3_ powder was dissolved in a mixed solution of 2.0 mL of anhydrous ethanol (CH_3_CH_2_OH, AR) and 1.5 mL of carbon disulfide (CS_2_), and then 2.0 mL of n‐butylamine (CH_3_(CH_2_)_3_NH_2_, GR 99.5%) was slowly added and stirred well. After that, every 2 mL of the above solution was diluted with 1 mL of anhydrous ethanol. Similarly, the C_60_ solution was prepared as follows: 5 mg of C_60_ powder was loaded into a 10 mL vial containing 2.0 mL of anhydrous ethanol (CH_3_CH_2_OH, AR) and 1.5 mL of carbon disulfide (CS_2_, GC), and then 2.0 mL of n‐butylamine (CH_3_(CH_2_)_3_NH_2_, GR 99.5%) was slowly added to the vial and stirred at room temperature. After stirring well, 2.16 mL of C_60_ solution was added to 1 mL of Sb_2_S_3_ precursor solution and stirred. Three hundred nanometers of Sb_2_S_3_ layers with and without C_60_ modification were deposited on commercial FTO glass, coated with a TiO_2_ layer, by spin coating at 3000 rpm for 60 s. Following the deposition, thin film was annealed on a hot plate in N_2_‐purge glove box at 100 °C for 1 min and 300 °C for 2 min. Sixty nanometers of Spiro‐OMeTAD was spin‐coated on Sb_2_S_3_ (C_60_)/TiO_2_/FTO substrate for 30 s at 3000 rpm as the hole transport layer. Solar cells were manufactured using the glass/FTO/TiO_2_/Sb_2_S_3_ (C_60_)/Spiro‐OMeTAD/Au structure. Gold contacts of 70 nm thickness and 0.09 cm^2^ area were deposited by thermal evaporation.

The surface morphology of bare and C_60_‐modified Sb_2_S_3_ thin films was characterized by SEM (FEI nova nano SEM450). XRD patterns of the films were characterized by XRD with Cu Kα1 (1.54056 Å) radiation (Bruker D8 Advance), and the optical properties of the films were measured using a spectrophotometer equipped with an integrating sphere (Perkin‐Elmer Lambda 950). In order to characterize the presence of C_60_ in the C_60_‐modified film, Raman measurements were performed using Raman spectrometer (Horiba Jobin Yvon, HR Evolution) equipped with the 532 nm line of the laser. Under standard test conditions (25 °C, AM 1.5, 100 mW cm^−2^), the *J*–*V* measurements were performed on bare and C_60_‐modified Sb_2_S_3_ thin film solar cells using an AM 1.5 solar simulator equipped with a 300 W xenon lamp (Model No. XES‐100S1, SAN‐EI, Japan). EQE of the bare and C_60_‐modified Sb_2_S_3_ film was obtained by Enlitech QER3011 system. Carrier transporting behavior of the device was characterized by EIS (PP211, ZAHNER, Germany) under appropriate open‐circuit voltage.

## Conflict of Interest

The authors declare no conflict of interest.
